# Effect of a care process programme on frail older people’s life satisfaction

**DOI:** 10.1002/nop2.300

**Published:** 2019-06-11

**Authors:** Helene Berglund, Susanne Gustafsson, Isabelle Ottenvall Hammar, Joel Faronbi, Synneve Dahlin‐Ivanoff

**Affiliations:** ^1^ The Frail Elderly Research Support Group (FRESH), Institute of Neuroscience and Physiology Sahlgrenska Academy at University of Gothenburg Gothenburg Sweden; ^2^ Institute of Health and Care Sciences Sahlgrenska Academy at University of Gothenburg Gothenburg Sweden; ^3^ The Gothenburg University Centre for Ageing and Health (AgeCap) Gothenburg Sweden; ^4^ Department of Occupational therapy and Physiotherapy Sahlgrenska University Hospital Gothenburg Sweden; ^5^ Department of Nursing Science, College of Health Science Obafemi Awolowo University Ile‐Ife Nigeria

**Keywords:** continuum of care, frail older adults, life satisfaction, outcome study

## Abstract

**Aim:**

The aim of this study was to analyse the effects of a full‐scale implementation of a care process programme on life satisfaction among frail older people, as compared with those receiving usual care.

**Design:**

The study includes participants from a full‐scale care process programme (*N = *77) and participants from a historical control group (*N = *66). The care process programme establishes a comprehensive continuum of care through components including case management, interprofessional teamwork and care‐planning meetings in the older people's own homes.

**Methods:**

Questionnaires were used and data were collected at baseline, with follow‐ups at three, six and 12 months.

**Results:**

The implementation of the full‐scale care process programme had a positive effect on life satisfaction among frail older people. From 6‐month–12‐month follow‐ups, a higher proportion of participants in the care process programme had positive life satisfaction outcomes, as compared with the historical control group.

## 1 INTRODUCTION

Frail older people often have several chronic impairments and increased vulnerability to stressors (Fried, Ferrucci, Darer, Williamson, & Anderson, 2004). They tend to have low degrees of well‐being and quality of life (Andrew, Fisk, & Rockwood, 2012; Bilotta et al., 2010; Gobbens & van Assen, 2014) and the negative connection between frailty and life satisfaction has been highlighted in previous research (Wilhelmson, Fritzell, Eklund, & Dahlin‐Ivanoff, 2013). Accordingly, it is important to investigate life satisfaction among frail older people.

## 2 BACKGROUND

Life satisfaction is closely connected to other similar concepts, such as subjective well‐being and quality of life. Subjective well‐being refers to the degree of well‐being according to people's own subjective evaluations of life. These evaluations involve judgements about life satisfaction and about pleasant as well as negative emotions (Diener, Oishi, & Ryan, 2013). It has been suggested that quality of life is a broader concept including both global and domain‐specific life satisfaction (Sirgy, 2012). Hence, life satisfaction can be considered as a subordinate concept in relation to subjective well‐being and quality of life. The concept of life satisfaction is especially useful for research in domains where questionnaires intended to measure quality of life are weak (Borg, Berg, Fugl‐Meyer, & Larsson, 2010), like the domain of social life.

Frail older people are frequently reliant on care from several various care providers. Deficient coordination among care providers leads to concerns for frail older people, highlighting the importance of introducing and realizing a comprehensive continuum of care (Cesari et al., 2016; Naylor & Berlinger, 2016). Previous studies on the outcomes of continuum of care interventions on frail older people's life satisfaction seem to be lacking. However, several studies have included evaluations of quality of life. A recent meta‐analysis showed that developed care‐planning services, consistent coordination among care providers and case management all had positive effects on quality of life among frail older people, especially with respect to general health and physical functioning (Chen, Tu, & Chen, 2017). Subjective well‐being has also been evaluated in several studies. In a systematic review by You, Dunt, Doyle, and Hsueh (2012), it was shown that studies evaluating the effect of coordination by a case manager and developed care planning revealed improved subjective well‐being for older people with different health problems, including disabilities. However, there are few studies examining the effects of such interventions on frail older people's life satisfaction.

In a previous larger intervention project, *Continuum of care for frail older people – a randomized controlled trail*, we developed several components to create a comprehensive continuum of care, including case management, interprofessional teamwork and organizing care‐planning meetings in the older people's own homes (Wilhelmson et al., 2011). We found positive results on frail older people's life satisfaction with respect to satisfaction with functional capacity and psychological health (Berglund, Hasson, Kjellgren, & Wilhelmson, 2015). The intervention also had a positive impact on older people's perceived quality of care (Berglund et al., 2013), frailty and independence in activities of daily living (Eklund, Wilhelmson, Gustafsson, Landahl, & Dahlin‐Ivanoff, 2013), as well as on self‐determination in daily life (Ekelund & Eklund, 2015). These measures were evaluated up to 12 months after baseline. Guided by the results, a full‐scale care process programme was developed and implemented, *From intervention trial (RCT) to full‐scale implementation research*, involving the same components as the intervention project (Wilhelmson et al., 2011). The implementation of the care process programme has shown positive results on frailty as well as self‐rated health (Ottenvall Hammar, Westgård, Eklund, Wilhelmson, & Dahlin‐Ivanoff, 2018).

In this study, we used data from the implementation of the full‐scale care process programme, as well as data from the historical control group in the previous intervention project (Wilhelmson et al., 2011). Consequently, we were able to evaluate whether our comprehensive continuum of care programme led to sustainable improvement in frail older people's life satisfaction. The aim of this study was to analyse the effects of a full‐scale implementation of a care process programme on life satisfaction among frail older people, as compared with those receiving usual care. We hypothesized that a care process programme, including coordination by a case manager, interprofessional teamwork and care‐planning meetings in the older people's own homes, might have a positive effect on frail older people's life satisfaction.

## 3 DESIGN

**Figure 1:**
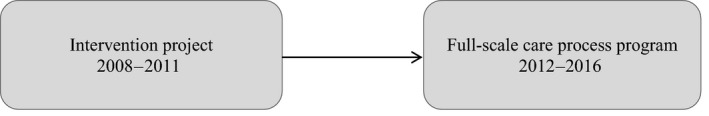
Timeframe of the intervention project and the full‐scale care process programme

This study includes participants from the full‐scale care process programme *From intervention trial (RCT) to full‐scale implementation research* (*N = *77) as well as participants from a historical control group who received usual care (*N* = 66). The historical control group was part of the previous intervention project *Continuum of care for frail older people – a randomized controlled trial* (Wilhelmson et al., 2011), which was a collaboration among municipal, primary and hospital care providers in a city with about 60,000 residents in Sweden. The implementation of the full‐scale care process programme took place in the same city, included further collaboration among the involved care organizations and comprised the same components and outcome measurements as did the previous intervention project. The timeframes of the previous intervention project and the full‐scale care process programme are illustrated in Figure 1.

## 4 METHODS

### 4.1 Care process programme and participants

The components of the care process programme were as follows:

Assessment of the need for health/social care and rehabilitation
Coordination by a case manager in the municipality
Assignment of an interprofessional team, including case manager, social worker, physiotherapist and/or occupational therapist
Support for relatives
Care‐planning meetings in the older person's own home (a few days after discharge if the person was hospitalized)
Follow‐up of needs and planned care

The historical control group in the previous intervention project received usual care, which did not include the assessment, the case manager or the care‐planning meeting in the older people's own home. Instead, if those in the control group needed new home care services, the care‐planning meeting took place at the hospital, which was the regular procedure. These meetings were conducted by hospital professionals and a regular municipal interprofessional team, including a social worker, a municipal nurse, an occupational therapist and/or a physiotherapist. No care‐planning meeting was held for those discharged to home directly after visiting the emergency department (Wilhelmson et al., 2011).

The participants in the care process programme consisted of a sample of people who had their 75th birthday during or before the study period. They were eligible if they sought care at an emergency department and thereafter were discharged to their own homes. Exclusion criteria were as follows: requiring acute medical services, clinically observed as having severe cognitive impairment or dementia and/or requiring palliative care as assessed by a nurse with geriatric competence.

The inclusion criteria in the previous intervention project were the same, but the age limit was 65 years or older. To make the participants in the previous intervention project comparable with the participants in the care process programme, we excluded those under 75 years. The eligibility for inclusion was the same as in the care process programme. Likewise, the exclusion criteria were the same in both the previous intervention project and the care process programme. The intention was to target frail older people at a high risk of future healthcare consumption (Wilhelmson et al., 2011).

### 4.2 Data collection

Baseline measurement was mostly made within a few days after hospital discharge or contact with the municipality and follow‐ups were performed at three, six and 12 months after the baseline measurement. The baseline and follow‐up data on the historical control group were collected between October 2008–December 2011. The data on the participants in the care process programme were collected between November 2012–March 2016. Data were collected via questionnaire. The questionnaire included a large amount of different scales and items about frailty, life satisfaction, activities of daily living and quality of care. The specific outcome measures in this study are described in the next section. Project assistants administered questionnaires orally during face‐to‐face interviews. All interviewers had experience with care of older people and performed the interviews according to established guidelines for each different outcome measure. The interviews took place in the older people's own homes. The questions and the response alternatives were read aloud to the participants. Sometimes the interviewers also showed participants the response alternatives on paper. The interviewers used text from a manual to thoroughly explain the scales and items in the questionnaire. The project leaders also organized meetings with all interviewers, during which the meaning of items was discussed in detail.

### 4.3 Outcome measures

Because our intention was to target frail older people, we used a set of frailty indicators, as recommended by Fried et al. (2004). Frailty was assessed as weakness, fatigue, weight loss, reduced physical activity, impaired balance, reduced gait speed, visual impairment and impaired cognition. The frailty indicators and measurement methods are described in detail in Wilhelmson et al. (2011). We deemed the older people to be frail if they fulfilled three or more frailty indicators (Fried et al., 2004).

The primary outcome measure in this study was life satisfaction. The validated LiSat‐11 scale was used as the measurement of older people's life satisfaction (Borg et al., 2010; Fugl‐Meyer, Bränholm, & Fugl‐Meyer, 1991). The scale comprises 11 items, including satisfaction with life as a whole, as well as satisfaction with work, financial situation, leisure, friends and acquaintances, sexual life, functional capacity, family life, partner relationship, physical health and psychological health. We excluded the items on satisfaction with work, sexual life, family life and partner relationship, as many of the older people did not consider these domains relevant to them. Each item has six response alternatives: “1‐very dissatisfied, 2‐dissatisfied, 3‐rather dissatisfied, 4‐rather satisfied, 5‐satisfied and 6‐very satisfied”. We dichotomized the response alternatives into not satisfied (score 1–4) and satisfied (score > 4), as recommended by Fugl‐Meyer et al. (1991) and Borg et al. (2010).

The LiSat‐11 scale has been validated in a representative sample of Swedish men and women aged 18–74 years. The item “life as a whole” correlated to all other items (Borg et al., 2010). The scale has adequate test–retest reliability, as well as discriminant and specificity validities (Fugl‐Meyer et al., 1991).

In this study, the number of items for which the participant gave a satisfied response was added together, at baseline and at each follow‐up. Consequently, this value varied between 0–7. We defined positive outcome on life satisfaction as the stability or increase of the overall satisfaction score between measurement points. We defined a negative outcome as the decrease of the value, or the stability of both values at 0 at two measurement points (indicating a persistent non‐satisfaction with all items).

## 5 ANALYSIS

A power calculation was done to ensure the possibility to compare results from the participants in the care process programme with the historical control group from the previous intervention project. The power calculation was based on results of the item on satisfaction with care planning. In the intervention project, 60% in the control group and 86% in the intervention group were very satisfied. To reach a power of 80% with alpha = 0.05 and a two‐sided test, 55 people were needed in each group.

Differences between the two groups regarding baseline characteristics were calculated using chi‐square tests (gender, cohabitant/living alone and education), Mann–Whitney *U* tests (frailty, life satisfaction) and *t* tests (age), respectively. The proportion of participants with a positive outcome on life satisfaction was compared for those in the care process programme and those in the historical control group. Three comparisons were made as follows: from baseline–3‐month follow‐up, 3‐month–6‐month follow‐ups and 6‐month–12‐month follow‐ups. The odds ratio (OR) was computed to compare outcomes between groups. A two‐sided *p* value of <0.05 and a 95% confidence interval (CI) served as indicators of statistical significance. Statistical analyses were carried out using SPSS statistical software package, version 19 for Windows (Chicago: SPSS Inc.).

We used intention‐to‐treat (ITT) strategies for managing missing data. This approach includes imputation of missing values (i.e., estimating what the value would be if it were not missing). Different strategies for imputation were used, according to the reason for the missing values (Committee for Medicinal Products for Human Use, 2010; Polit & Gillespie, 2010). The median change of deterioration (MCD) in life satisfaction was computed for baseline–three‐month follow‐up, three‐month–six‐month follow‐ups and six‐month–12‐month follow‐ups for participants who completed the follow‐ups. The MCD was imputed for participants who declined to continue before any of the follow‐ups, as proposed in earlier studies (Eklund et al., 2013; Gustafsson et al., 2012). This approach was based on the assumption that participants who declined to continue had deteriorated health. For participants who died before any of the follow‐ups, worst‐case imputation was done (Eklund et al., 2013; Gustafsson et al., 2012; Polit & Gillespie, 2010). For participants who completed the study but did not have reported values at any of the measurement points, as well as for internal loss, we used the participants’ own obtained values at a different point in time (Berglund et al., 2015; Engels & Diehr, 2003; Polit, 2010). In one case in the historical control group and two cases in the care process programme, imputations were not possible due to missing data on all measurement points. Additionally, as a sensitivity analysis, we compared the ITT analyses with complete case (CC) analyses, showing aligned trends (results not shown).

## 6 ETHICS

All participants signed a written, informed consent form. The previous intervention project was approved by the Regional Ethical Review Board in Gothenburg, Sweden, registration number 413–08. It was registered at Clinical Trials Gov: NCT01260493. Additional ethical approval was obtained for the full‐scale care process programme, registration number T140‐12.

## 7 RESULTS

### 7.1 Participants and drop‐outs

**Figure 2:**
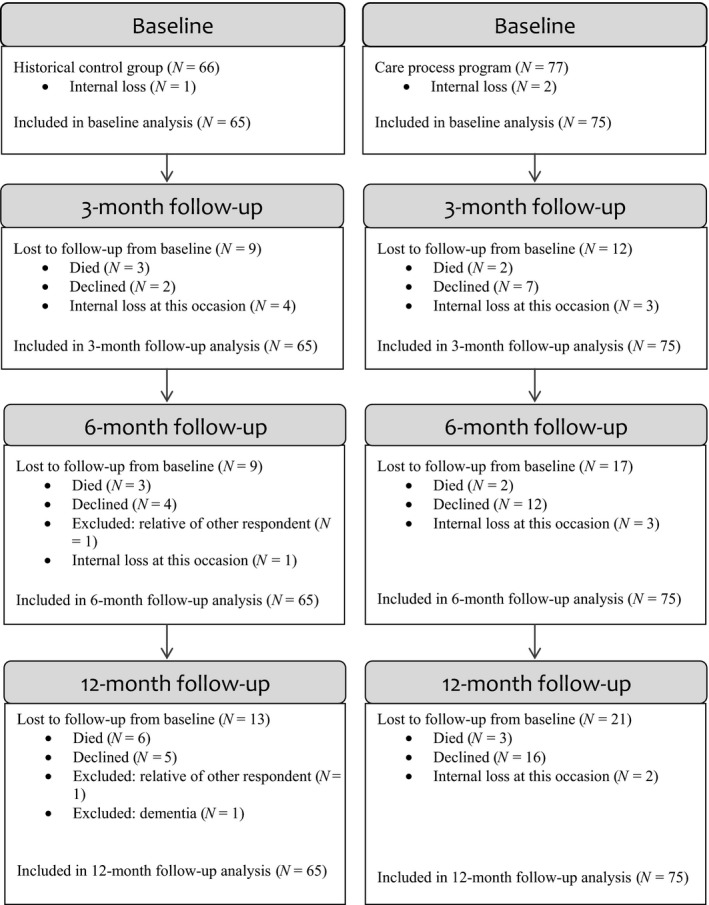
Participants and drop‐outs in the study

Baseline data were collected from 143 older people: 66 from the historical control group from the intervention project and 77 from the care process programme. In the historical control group, approximately 14% (*N = *9) dropped out from baseline–three months, 14% (*N = *9) from baseline–six months and 20% (*N = *13) from baseline–12 months. In the care process programme, 16% (*N = *12) had dropped out at three months, 22% (*N = *17) at six months and 27% (*N = *21) at 12 months. The differences in drop‐outs between the two groups were non‐significant at all follow‐ups. Details on drop‐outs and participants included in the present analysis are presented in Figure 2.

Baseline characteristics of participants are presented in Table 1. There were no statistically significant differences between the groups for cohabitant/living alone, education, age, frailty or life satisfaction. However, there were significantly more women in the care process programme.

**Table 1:** Gender, cohabitant/living alone, education, age, frailty and life satisfaction for participants in historical control group and care process programme at baseline (*N = *143)

	Historical control group *n* (%)	Care process programme *n* (%)	*p* value between groups
Female	36 (55)	60 (78)	0.01**
Living alone	39 (59)	51 (66)	0.48
Elementary school or less	32 (49)	33 (43)	0.66
Age	Mean (range)	Mean (range)	0.99
	84 (77–92)	84 (75–100)	
Frailty^a^	Median (range)	Median (range)	0.77
	3 (1–7)	4 (0–7)	
Life satisfaction^b^ (*N = *140)	Median (range)	Median (range)	0.65
	3 (0–7)	4 (0–7)	

a Number of fulfilled frailty indicators, including weakness, fatigue, weight loss, reduced physical activity, impaired balance, reduced gait speed, visual impairment and impaired cognition. In a few cases, values were imputed, according to documentation in medical journal.

b Number of items scored >4 (satisfied).

** *p* value is less than 0.01.

Both drop‐outs and participants who completed all follow‐ups were compared with respect to baseline characteristics at three, six and 12 months. There were no statistically significant differences for gender, cohabitant/living alone, education and age at any of the follow‐ups. For frailty, there were no statistically significant differences at six‐month follow‐up, but there were more frailty indicators among the drop‐outs compared with the participants at three months (*p* = 0.01) and 12 months (*p* = 0.05).

### 7.2 Life satisfaction

There were no statistically significant differences between the historical control group and care process programme on life satisfaction from baseline–three‐month follow‐up or from 3–6‐month follow‐ups. However, from 6–12‐month follow‐ups there were higher proportions of participants with positive outcome on life satisfaction in the care process programme (OR 2.99, CI 1.50–5.98) (Table 2).

**Table 2:** Proportion (%), odds ratio (OR) and 95% confidence Interval (CI) for positive outcome on life satisfaction between baseline–3‐month follow‐up, 3–6‐month follow‐ups and 6–12‐month follow‐ups (*N = *140)

	Historical control group		Care process programme		95% CI
	%	OR	%	OR	
Baseline–3 months	66	1	55	0.62	0.31–1.23
3–6 months	60	1	64	1.19	0.60–2.35
6–12 months	43	1	69	2.99	1.50–5.98

## 8 DISCUSSION

A full‐scale care process programme targeting frail older people was implemented and its impact on the older people's life satisfaction was evaluated. The programme included case management, interprofessional teamwork and organizing of care‐planning meetings in the older people's own homes. From baseline–three‐month follow‐up and from 3–6‐month follow‐ups, no effect could be seen on life satisfaction. However, from 6–12‐month follow‐ups, there was a significantly higher proportion of participants with positive outcome on life satisfaction in the care process programme compared with the historical control group. Frail older people in the care process programme were about three times more likely to improve or maintain life satisfaction.

Evaluations of life satisfaction seem to be rare in previous programmes targeting frail older people. However, similar programmes have shown positive outcomes with respect to quality of life (Chen et al., 2017). A recent intervention study involving comprehensive assessment, case management and interprofessional teamwork and care planning, showed a moderate, significant effect on frail older people's quality of life after 12 months (Looman, Fabbricotti, de Kuyper, & Huijsman, 2016). Likewise, subjective well‐being has been shown to be improved by programmes including case management and developed care planning, according to a systematic review by You et al. (2012).

In the present study, there were no differences in life satisfaction between the participants in the historical control group and the care process programme from baseline–the three‐month follow‐up or from 3–6‐month follow‐ups. The difference in favour of the care process programme was detected later in time, between 6–12‐month follow‐ups. Similarly, in the previous intervention project, an effect on life satisfaction was seen between 6–12‐month follow‐ups, in favour of the intervention (Berglund et al., 2015). The intervention project and the full‐scale care process programme included case management, interprofessional teamwork and organizing of care‐planning meetings in the older people's own homes. It might be that the older people felt the full impact of all these components in the programme only after some time. Hence, the result of the previous intervention project can be confirmed by this study and the care process programme seems to be sustainable over time. Our result implies that the implementation of the different components in a real context has the same impact as when they were performed in an intervention project.

The positive result on life satisfaction might be explained by the assumption that the older people felt secure and comfortable with the programme. The care‐planning meetings were held in the older people's own homes, a more familiar and comfortable environment than the hospital. This is supported by the evaluation of the previous intervention project's impact on the older people's views of the quality of care. They perceived higher quality of care planning when it was performed in their own homes (Berglund et al., 2013). The case manager probably also contributed to security and comfort for the older people. It was shown in the previous project that the older people in the intervention group had better knowledge of whom to contact, up to 12 months after baseline (Berglund et al., 2013). In total, this probably led to the older people's greater satisfaction with care, which in turn resulted in higher life satisfaction.

There is an urgent need for further development of programmes targeting frail older people. The implementation of a combination of components in the present programme positively correlated with the older people's life satisfaction, which is a key aspect of their well‐being. Prince et al. (2015) stressed that age‐appropriate care services need immediate attention to meet the needs of the growing population of frail older people and to improve their conditions. The implementation of comprehensive assessment and improved coordination of care are key priorities in this development.

### 8.1 Methodological considerations

The primary strength of this study is the inclusion of frail older people, who are often difficult to involve in longitudinal studies. The older people in this study were followed up to one year, with moderate drop‐outs. Longitudinal research is especially valuable for evaluating the benefits of a care process programme, which is important because there is an emerging need to prioritize the care of frail older people and to organize a comprehensive continuum of care. Interprofessional teamwork and case management are essential components in the continuum of care for frail older people (Cesari et al., 2016).

We compared baseline characteristics for participants who completed all follow‐ups and those who dropped out at three, six and 12 months. There were more frailty indicators at the three‐ and 12‐month follow‐ups in the drop‐outs as compared with participants. This supports our imputation strategies for missing values for participants who declined or died, because it implies that the drop‐outs were not random. The older people who declined likely thought the interviews were too demanding, due to deteriorated health.

A limitation in this study concerns the measurement of the older people's life satisfaction. We excluded four of the items in the LiSat‐11 scale, because many of the older people did not consider them to be relevant to their lives. The exclusion of these items could have influenced the internal consistency of the test. However, if we had used all items, there might have been several participants with internal missing at baseline and the follow‐ups causing a larger number of drop‐outs and a risk of bias. If one or more items had been missing at all measurement points, we would not have been able to use these participants’ data at all, because we used the sum of items for which the participants were satisfied in the analysis.

Another limitation might be the presence of a confounding health‐related factor influencing life satisfaction among the participants. However, this is quite unlikely as the outcome measures covered a range of different health‐related factors, involving all frailty indicators. There were no differences between the groups at baseline for the amount of fulfilled frailty indicators.

Moreover, an additional limitation might be that there were more women in the care process programme than in the historical control group at baseline. We did not adjust the analysis to control for this because women and men probably do not differ in life satisfaction. Previous research has shown that gender seems not to influence life satisfaction (Berglund, Hasson, Wilhelmson, Dunér, & Dahlin‐Ivanoff, 2016).

## 9 CONCLUSIONS AND RELEVANCE TO CLINICAL PRACTICE

The implementation of a full‐scale care process programme for frail older people, including case management, interprofessional teamwork and care‐planning meetings in the older people's own homes, had some benefits for their life satisfaction. Frail older people constitute most of the people in need of care and support. In clinical practice, healthcare professionals need to account for care process programmes’ impact on life satisfaction. Because life satisfaction is of great importance for frail older people's well‐being, we recommend that policymakers and managers in care of older people further promote programmes involving a comprehensive continuum of care.

## CONFLICT OF INTEREST

The authors declare that they have no conflict of interests.

## AUTHOR CONTRIBUTIONS

Helene Berglund made substantial contributions to conception and design, made the statistical analyses, drafted the manuscript, gave final approval of the version to be published and agreed to be accountable for all aspects of the work. Susanne Gustafsson, Isabelle Ottenvall Hammar and Joel Faronbi made substantial contributions to conception and design, revised the manuscript critically for intellectual content, gave final approval of the version to be published and agreed to be accountable for all aspects of the work. Synneve Dahlin‐Ivanoff made substantial contributions to conception and design, gave statistical advice, revised the manuscript critically for intellectual content, gave final approval of the version to be published and agreed to be accountable for all aspects of the work.

## References

[nop2300-bib-0001] Andrew, M. K. , Fisk, J. D. , & Rockwood, K. (2012). Psychological well‐being in relation to frailty: A frailty identity crisis? International Psychogeriatrics, 24, 1347–1353. 10.1017/S1041610212000269 22436131

[nop2300-bib-0002] Berglund, H. , Hasson, H. , Kjellgren, K. , & Wilhelmson, K. (2015). Effects of a continuum of care intervention on frail older persons’ life satisfaction: A randomized controlled study. Journal of Clinical Nursing, 24, 1079–1090. 10.1111/jocn.12699 25293644

[nop2300-bib-0003] Berglund, H. , Hasson, H. , Wilhelmson, K. , Dunér, A. , & Dahlin‐Ivanoff, S. (2016). The impact of socioeconomic conditions, social networks and health on frail older people’s life satisfaction: A cross‐sectional study. Health Psychology Research, 4, 5578 10.4081/hpr.2016.5578 27403463 PMC4926029

[nop2300-bib-0004] Berglund, H. , Wilhelmson, K. , Blomberg, S. , Dunér, A. , Kjellgren, K. , & Hasson, H. (2013). Older people's views of quality of care: A randomised controlled study of continuum of care. Journal of Clinical Nursing, 22, 2934–2944. 10.1111/jocn.12276 23808647

[nop2300-bib-0005] Bilotta, C. , Bowling, A. , Case, A. , Nicolini, P. , Mauri, S. , Castelli, M. , & Vergani, C. (2010). Dimensions and correlates of quality of life according to frailty status: A cross‐sectional study on community‐dwelling older adults referred to an outpatient geriatric service in Italy. Health and Quality of Life Outcomes, 8, 56 10.1186/1477-7525-8-56 20529325 PMC2889875

[nop2300-bib-0006] Borg, T. , Berg, P. , Fugl‐Meyer, K. , & Larsson, S. (2010). Health‐related quality of life and life satisfaction in patients following surgically treated pelvic ring fractures. A prospective observational study with two years follow‐up. Injury, 41, 400–404. 10.1016/j.injury.2009.11.006 20005513

[nop2300-bib-0007] Cesari, M. , Prince, M. , Thiyagarajan, J. A. , De Carvalho, I. A. , Bernabei, R. , Chan, P. , … Vellas, B. (2016). Frailty: An emerging public health priority. Journal of the American Medical Directors Association, 17, 188–192. 10.1016/j.jamda.2015.12.016 26805753

[nop2300-bib-0008] Chen, H. M. , Tu, Y. H. , & Chen, C. M. (2017). Effect of continuity of care on quality of life in older adults with chronic diseases: A meta‐analysis. Clinical Nursing Research, 26, 266–284. 10.1177/1054773815625467 26790451

[nop2300-bib-0009] Committee for Medicinal Products for Human Use (2010). Guideline on missing data in confirmatory clinical trials. London, United Kingdom: European Medicines Agency.

[nop2300-bib-0010] Diener, E. , Oishi, S. , & Ryan, K. L. (2013). Universals and cultural differences in the causes and structure of happiness: A multilevel review In Keyes C. L. M. (Ed.), Mental well‐being: International contributions to the study of positive mental health (pp. 153–176). Dordrecht, The Netherlands: Springer 10.1007/978-94-007-5195-8_8

[nop2300-bib-0011] Ekelund, C. , & Eklund, K. (2015). Longitudinal effects on self‐determination in the RCT “Continuum of care for frail elderly people”. Quality in Ageing and Older Adults, 16, 165–176. 10.1108/QAOA-12-2014-0045

[nop2300-bib-0012] Eklund, K. , Wilhelmson, K. , Gustafsson, H. , Landahl, S. , & Dahlin‐Ivanoff, S. (2013). One‐year outcome of frailty indicators and activities of daily living following the randomised controlled trial;“Continuum of care for frail older people”. BioMed Central Geriatrics, 13, 76 10.1186/1471-2318-13-76 23875866 PMC3750658

[nop2300-bib-0013] Engels, J. M. , & Diehr, P. (2003). Imputation of missing longitudinal data: A comparison of methods. Journal of Clinical Epidemiology, 56, 968–976. 10.1016/S0895-4356(03)00170-7 14568628

[nop2300-bib-0014] Fried, L. P. , Ferrucci, L. , Darer, J. , Williamson, J. D. , & Anderson, G. (2004). Untangling the concepts of disability, frailty and comorbidity: Implications for improved targeting and care. The Journals of Gerontology Series A: Biological Sciences and Medical Sciences, 59, 255–263. 10.1093/gerona/59.3.M255 15031310

[nop2300-bib-0015] Fugl‐Meyer, A. R. , Bränholm, I. B. , & Fugl‐Meyer, K. S. (1991). Happiness and domain‐specific life satisfaction in adult northern Swedes. Clinical Rehabilitation, 5, 25–33. 10.1177/026921559100500105

[nop2300-bib-0016] Gobbens, R. J. J. , & van Assen, M. A. L. M. (2014). The prediction of quality of life by physical, psychological and social components of frailty in community‐dwelling older people. Quality of Life Research, 23, 2289–2300. 10.1007/s11136-014-0672-1 24671672

[nop2300-bib-0017] Gustafsson, S. , Eklund, K. , Wilhelmson, K. , Edberg, A.‐K. , Johansson, B. , Kronlof, G. H. , … Dahlin‐Ivanoff, S. (2012). Long‐term outcome for ADL following the health‐promoting RCT—Elderly Persons in the Risk Zone. The Gerontologist, 53, 654–663. 10.1093/geront/gns121 22936539

[nop2300-bib-0018] Looman, W. M. , Fabbricotti, I. N. , de Kuyper, R. , & Huijsman, R. (2016). The effects of a pro‐active integrated care intervention for frail community‐dwelling older people: A quasi‐experimental study with the GP‐practice as single entry point. BioMed Central Geriatrics, 16, 43 10.1186/s12877-016-0214-5 26879893 PMC4755064

[nop2300-bib-0019] Naylor, M. , & Berlinger, N. (2016). Transitional Care: A Priority for Health Care Organizational Ethics. Hastings Center Report, 46, S39–S42. 10.1002/hast.631. 27649919

[nop2300-bib-0020] Ottenvall Hammar, I. , Westgård, T. , Eklund, K. , Wilhelmson, K. , & Dahlin‐Ivanoff, S. (2018). From Intervention Trial to Full‐scale Implementation Research: Positive Tendencies for Frailty and Self‐rated Health in Frail Older People. Inernational Journal of Geriatrics and Gerontology: IJGG‐112, 10.29011/2577-0748.180012.

[nop2300-bib-0021] Polit, D. F. (2010). Statistics and data analysis for nursing research, 2nd ed. Boston, MA: Pearson.

[nop2300-bib-0022] Polit, D. F. , & Gillespie, B. M. (2010). Intention‐to‐treat in randomized controlled trials: Recommendations for a total trial strategy. Research in Nursing & Health, 33, 355–368. 10.1002/nur.20386 20645423

[nop2300-bib-0023] Prince, M. J. , Wu, F. , Guo, Y. , Gutierrez Robledo, L. M. , O'Donnell, M. , Sullivan, R. , & Yusuf, S. (2015). The burden of disease in older people and implications for health policy and practice. Lancet, 385, 549–562. 10.1016/S0140-6736(14)61347-7 25468153

[nop2300-bib-0024] Sirgy, M. J. (2012). The psychology of quality of life: Hedonic well‐being, life satisfaction and eudaimonia. Dordrecht, the Netherlands: Springer, 10.1007/978-94-007-4405-9

[nop2300-bib-0025] Wilhelmson, K. , Duner, A. , Eklund, K. , Gosman‐Hedström, G. , Blomberg, S. , Hasson, H. , … Dahlin‐Ivanoff, S. (2011). Continuum of care for frail elderly people: Design of a randomized controlled study of a multi‐professional and multidimensional intervention targeting frail elderly people. BioMed Central Geriatrics, 11, 24 10.1186/1471-2318-11-24 21569570 PMC3118103

[nop2300-bib-0026] Wilhelmson, K. , Fritzell, E. , Eklund, K. , & Dahlin‐Ivanoff, S. (2013). Life satisfaction and frailty among older adults. Health Psychology Research, 1, e32 10.4081/hpr.2013.1515 26973917 PMC4768568

[nop2300-bib-0027] You, E. C. , Dunt, D. , Doyle, C. , & Hsueh, A. (2012). Effects of case management in community aged care on client and carer outcomes: A systematic review of randomized trials and comparative observational studies. BioMed Central Health Services Research, 12, 395 10.1186/1472-6963-12-395 23151143 PMC3508812

